# On your bike! a cross-sectional study of the individual, social and environmental correlates of cycling to school

**DOI:** 10.1186/1479-5868-8-123

**Published:** 2011-11-10

**Authors:** Georgina SA Trapp, Billie Giles-Corti, Hayley E Christian, Max Bulsara, Anna F Timperio, Gavin R McCormack, Karen P Villaneuva

**Affiliations:** 1 Centre for the Built Environment and Health, School of Population Health, The University of Western Australia, Perth (6009), Australia; 2 Institute of Health and Rehabilitation, University of Notre Dame, Perth (6959), Australia; 3 Centre for Physical Activity and Nutrition Research, Deakin University, Victoria (3125), Australia; 4 Population Health Intervention Research Centre, Department of Community Health Sciences, University of Calgary, Alberta (T2N 4Z6). Canada

**Keywords:** Cycling, children, active school transport, physical activity

## Abstract

**Background:**

Active school transport (AST) has declined rapidly in recent decades. While many studies have examined walking, cycling to school has received very little attention. Correlates of cycling are likely to differ to those from walking and cycling enables AST from further distances. This study examined individual, social and environmental factors associated with cycling to school among elementary school-aged children, stratified by gender.

**Methods:**

Children (n = 1197) attending 25 Australian primary schools located in high or low walkable neighborhoods, completed a one-week travel diary and a parent/child questionnaire on travel habits and attitudes.

**Results:**

Overall, 31.2% of boys and 14.6% of girls cycled ≥ 1 trip/week, however 59.4% of boys and 36.7% of girls reported cycling as their preferred school transport mode. In boys (but not girls), school neighborhood design was significantly associated with cycling: i.e., boys attending schools in neighborhoods with high connectivity and low traffic were 5.58 times more likely to cycle (95% CI 1.11-27.96) and for each kilometer boys lived from school the odds of cycling reduced by 0.70 (95% CI 0.63-0.99). Irrespective of gender, cycling to school was associated with parental confidence in their child's cycling ability (boys: OR 10.39; 95% CI 3.79-28.48; girls: OR 4.03; 95% CI 2.02-8.05), parental perceived convenience of driving (boys: OR 0.42; 95% CI 0.23-0.74; girls: OR 0.40; 95% CI 0.20-0.82); and child's preference to cycle (boys: OR 5.68; 95% CI 3.23-9.98; girls: OR 3.73; 95% CI 2.26-6.17).

**Conclusion:**

School proximity, street network connectivity and traffic exposure in school neighborhoods was associated with boys (but not girls) cycling to school. Irrespective of gender, parents need to be confident in their child's cycling ability and must prioritize cycling over driving.

## Introduction

Physically active children are less likely to develop chronic disease risk factors [1], more likely to experience enhanced mental and emotional wellbeing [2,3] and more likely to remain active during adolescence and adulthood [4]. Participation in active school transport (AST) has the potential to improve health through its contribution to overall physical activity levels and fitness. For example, positive associations between cycling to school and cardiovascular fitness have been found among children [5-7]. Despite the health, economic and environmental benefits of AST, levels of AST have declined rapidly in recent decades. For example, US National Personal Transportation Survey data show that the proportion of students who walk or cycle to school fell from 40.7% in 1969 to just 12% in 2001 [8]. Although not as pronounced, National Travel Survey data from the UK collected between 1975/76 and 2009 also showed reductions in the proportion of adolescents (11-16 years of age) walking (53 to 38%) and cycling (7 to 3%) to school [9]. In addition, from 1985 to 2001, the prevalence of walking and cycling to school among Australian children (9-13 years of age) declined by 50% and 80%, respectively [10]. It is therefore important to investigate correlates of AST to inform efforts to increase this important form of physical activity. While several studies have examined correlates of AST [11-14], the literature focuses mainly on walking, with little attention given to cycling. As a result, there is a lack of empirical knowledge about factors relating specifically to cycling to school. Correlates of cycling are likely to differ to those from walking and cycling enables AST from further distances.

To date, three studies have examined correlates of cycling to school. Positive associations found between individual-level correlates and cycling to school include: being older or male [15], and having the skills and ability to safely ride a bicycle [15]. Positive associations found between social correlates and cycling to school have included: social support from family and friends [16], parental perceived inconvenience of driving child to school [16] and parental absence at home before or after school [16,17]. Cycling to school has also been positively associated with physical environmental features including living close to the school [16,17], residing in a metropolitan area [15] and perceptions of; low/safe traffic [15,16], appropriate lighting and weather conditions [15], a safe neighborhood or route to school [16], neighborhood sense of community [16] and high walkability [16].

Limited evidence exists to guide public health policy and interventions developed to encourage cycling among children. To the best of our knowledge, only one study has taken an ecological [18] approach - simultaneously examining individual, social, **and **environmental correlates of cycling to school among children - and no study has stratified by gender, despite girls being less likely than boys to cycle to school [17,19]. Moreover, few objectively-assessed built environmental variables (and potential mediators) [20] have been examined in relation to cycling to school among children. Thus, the objective of this explorative study was to investigate individual, social, and environmental correlates of cycling to school among boys and girls using an ecological framework.

## Methods

This paper draws data from the TRavel, Environment and Kids (TREK) project [21]. Cross-sectional data were collected in 2007 using self-report travel diaries, self-completed questionnaires (child and parent), anthropometric measurements and Geographic Information Systems (GIS). The University of Western Australia's Human Ethics Committee provided ethics approval (RA/4/1/1394).

### School selection and school walkability index

Methods used to select schools have been described fully elsewhere [21]. In brief, the walkability of areas within 2 km of all schools in metropolitan Perth, Western Australia (n = 238) was assessed using two GIS measures, pedsheds and road traffic volume (RTV). Pedshed, a measure of connectivity, was computed by taking the ratio of the pedestrian network area within 2 km to the Euclidian distance area (2 km crow-fly buffer). RTV was the total length of all roads within the 2 km crow-fly buffer (excluding access roads) divided by the total length of access roads (designed to carry < 3000 vehicles/day). Deciles of each measure were created and used to create a school-specific walkability index (SWI) for each school. Schools were coded into six groups based on their walkability and socioeconomic (SES) status (i.e., high walkability and either high/med/low SES or low walkability and either high/medium/low SES) and the four top-ranking schools in each category were invited to participate (excluding those located in semi-rural areas or in high walkable areas but located on a busy road) until four schools were recruited within each category (69% response rate). Due to small numbers, one additional school in the high walkability, low SES category was recruited (n = 25 schools).

### Participant selection

One class from grade 5, 6 and 7 in each school was randomly selected to participate until a minimum of 30 children per grade were recruited. Informed written consent was obtained from all children and their parents. In total, 1480 children (57% response rate (RR)) and 1314 of their parents (88.8% RR) participated. Children completed a questionnaire in class guided by research staff and parents completed a questionnaire at home.

### Cycling behavior

Children kept a 5-day travel diary indicating their mode of transport to and from school on each day of the school survey week. This was a modified version of a travel diary previously validated in children of similar ages (9-11 year olds) [22] and was pilot tested on 160 10-12 year old children. Children were dichotomized into ≥ 1 cycle trips/week versus no cycle trips/week.

### Questionnaire development

Test-retest reliability (1 week) of survey items was assessed (4 schools; n = 160 10-12 year old children; n = 101 parents) and items with acceptable reliability (i.e., kappa or ICC > 0.60) were included in the final survey with items < 0.60 modified to enhance reliability. A table given later in the results section presents the ICC and kappa values of all items.

### Individual factors

Eleven single items measuring individual factors were examined. Height and weight were measured with calibrated digital scales and portable stadiometers with the children dressed in light clothing and no shoes. Body Mass Index (BMI, kg/m^2^) estimates were collapsed into age and sex-specific categories (i.e., acceptable weight, overweight and obese) based on internationally-recognized cut-points [23].

### Social factors

Thirteen single items and three scales measuring social factors were examined using self- and parent-report measures. The three scales were created from nine single items using factor analysis and a varimax rotation i.e., a "peer support" scale ("my friends like to ride a bike to get to places" and "my friends think it is cool to ride a bike to school", Cronbach's α = 0.627); a "disapproval from others" scale (i.e., "if I allowed my child to ride a bike to school with other children but without an adult present:" "other parents would disapprove", "the school principal would be concerned", "teachers at the school would be concerned" and "members of my family would be concerned", Cronbach's α = 0.895); and a "fear of stranger danger" scale (i.e., "how fearful are you that if your child walked or cycled in your neighborhood without an adult he or she may:" "be approached on the street by a stranger", "be taken by a stranger", "be hurt by a stranger", Cronbach's α = 0.935).

### Perceived environmental factors

Fourteen single items measuring perceived environmental factors were examined using child and parent-report measures.

### Objective environmental factors

As noted above, the SWI incorporated two indices (i.e., pedshed and RTV). The pedshed and RTV indices were dichotomized into high and low categories. School SES was based on an unpublished index used by the Western Australian Department of Education and Training that provides a measure of the socio-economic indicator for the school. This area-level SES index is based on parent occupation, parent level of education attainment, income level of parents, family structure (single parent family, etc.), language spoken at home, tenancy (home ownership or renting, etc.), crowding (number of occupants of a dwelling) and Aboriginality. The information is gathered from the national census and double weighting is given to the factors of occupation, income and education. The SES index was collapsed into tertiles (i.e. low, medium or high SES). The shortest distance (in meters) along the *pedestrian network *(i.e., formal street network plus informal networks such as laneways, walkways, pedestrian access ways at the end of cul-de-sacs and paths through parks) was calculated from each child's home address (parent reported) to the 'access point' of the school boundary (i.e. school polygon) using GIS.

### Statistical analyses

Children with no matching parent questionnaire (n = 183) and those who did not own a bicycle (n = 101) were excluded. There were no significant demographic differences between those excluded and those included in the sample. Independent *t *tests and Pearson's Chi-square were used to examine gender differences in sample characteristics. Each of the individual, social and environmental variables were tested for bivariate associations with the outcome variable (i.e., cycles ≥ 1 trip versus no trips/week to or from school) and non-significant variables (*p *> 0.1) were excluded from further analyses. Multivariate logistic regression analyses was undertaken and significant variables (*p *≤ 0.05) remained in the final model to estimate the odds of cycling to or from school ≥ 1 trip/week. Four blocks of independent variables - objective environment (model 1), perceived environment (model 2), social factors (model 3) and individual factors (model 4) - were sequentially added to the model. All models adjusted for school clustering, the child's grade and highest level of maternal education and were stratified by gender. Effect modification by pedshed and RTV was also examined. Mediation analyses were undertaken using the Baron and Kenny approach [24]. SPSS 14 and Stata/IC 11.0 were used for analysis (2010). All independent variables were checked for effects of multicollinearity.

## Results

### Sample characteristics

Demographic characteristics of boys and girls were similar (*p *> 0.05), although commuting behavior (i.e., cycling, walking, and driving) and transport mode preference significantly differed (Table 1). Approximately 20.0% of children were overweight and 3.1% obese. The majority of parent participants were female (87.9%) with at least a secondary/trade/diploma level of education (51.3%). Children resided, on average, 1.7 km (SD = 2.4) from the school. A greater proportion of boys cycled to school than girls (1.8 vs. 0.8 trips/week, *p *< 0.001), whilst a greater proportion of girls walked (3.3 vs. 2.8 trips/week, *p *= 0.045) or were driven (5.6 vs. 5.0 trips/week, *p *= 0.010) than boys. Although only 31.2% of boys and 14.6% of girls cycled at least one trip to school, double the number of boys (59.4%) and more than twice the number of girls (36.7%) reported cycling as their preferred mode of transport to school.

**Table 1:** Sample characteristics stratified by gender

	Gender	
	
Sample Characteristic	Boys (n = 573)	Girls (n = 624)
**Child's grade level (%)**		
5	29.7	25.8
6	35.8	37.3
7	34.6	36.9
**Weight status of child (%)**		
Acceptable	67.5	58.0
Overweight or Obese	19.7	17.9
Refused	12.7	24.0
**Socio-economic status of school (%)**		
Low	22.2	26.8
Medium	36.5	34.3
High	41.4	38.9
**School walkability index (%)**		
Low	54.5	51.9
High	45.5	48.1
**Sex of responding parent (%)**		
Male	13.8	10.6
Female	86.2	89.4
**Maternal education (%)**		
Less than Secondary	26.6	29.6
Secondary/trade/diploma	55.1	54.0
Bachelor degree or higher	18.3	16.4
**Maternal employment (%)**		
None	24.2	28.1
Part-time	47.3	48.5
Full-time	28.5	23.4
**Distance to school**		
**(mean km [SE])**	1.8 (0.1)	1.6 (0.1)
**Commuting to/from school**		
**(mean trips/week, [SE])**		
Cycles	1.8 (0.1)***	0.8 (0.1)
Walks	2.8 (0.2)*	3.3 (0.2)
Driven	5.0 (0.2)**	5.6 (0.2)
**Cycles to/from school ≥ 1 trip/week (%)**	31.2***	14.6
**Child's preference is to cycle (%)**	59.4***	36.7

*p ≤ 0.05; **p ≤ 0.01; *** *p *≤ 0.001, SE = standard error

Table 2 presents bivariate associations between cycling at least 1 school trip/week and individual, social and physical environmental variables stratified by gender. For boys, 10 individual-level, 13 social, and 10 physical environmental factors were eligible (*p *≤ 0.10) for inclusion into the multivariate logistic regression models. For girls, eight individual-level, seven social, and nine physical environmental factors were eligible (*p *≤ 0.10) for inclusion.

**Table 2:** Bivariate associations of cycling to/from school ≥ 1 trip/week with individual, social and environmental variables

		Boys		Girls	
		
		Cycles ≥ 1 trip/week		Cycles ≥ 1 trip/week	
			
Variables	Item Reliability^Φ^	% No (n = 394)	% Yes (n = 179)	% No (n = 533)	% Yes (n = 91)
**Individual factors**					
***Family factors***					
Maternal education^§^	0.96 (97.6%)^‡^				
- Less than Secondary		25.0	30.0	**30.3**	**25.0^#^**
- Secondary/trade/diploma		56.3	52.4	**52.2**	**65.5**
- Bachelor degree or higher		18.7	17.6	**17.5**	**9.5**
Maternal employment^§^	0.92 (96.4%)^‡^				
- None		**21.6**	**29.6^#^**	29.2	20.3
- Part-time		**50.2**	**41.4**	47.3	56.5
- Full-time		**28.2**	**29.0**	23.5	23.2
Adult home after school on most days^§^	0.32 (91.6%)^‡^	**80.1**	71.9*	**83.6**	72.6*
Scheduling commitments before/after school^§^	0.56 (79.3%)^‡^	**40.6**	21.6***	37.6	28.7
***Child factors & perceptions***					
Overweight or obese	-	**20.2**	**27.8^#^**	23.8	23.0
Cycling is child's preferred school transport mode^¥^	0.61 (80.5%)^‡^	**47.8**	85.1***	**30.7**	71.4***
Cycling to school is cool^¥a^	0.62^ж^	**50.8**	73.7***	**47.9**	64.8**
Cycling to school is more convenient^¥a^	0.67^ж^	**45.2**	74.9***	**36.8**	68.1***
Confident in ability to cycle to school without adult^¥a^	0.67^ж^	**81.7**	96.6***	**73.1**	87.9**
Feels safer being driven to school than cycling^¥a^	0.60^ж^	**29.2**	14.0***	**30.3**	19.8*
Would feel safe whilst cycling to school^¥a^	0.68^ж^	**50.0**	67.0***	**37.0**	50.5*
**Social factors**					
***Parent perceptions***^b^					
Very/extremely fearful child may be injured if they cycled to school without adult^§c^	0.62^ж^	**22.7**	11.2**	26.9	18.6
Very/extremely fearful of stranger danger^§c^	0.91^ж^	**87.6**	79.9*	**90.3**	75.6***
Often sees/hears news items about traffic dangers ^§c^	0.40^ж^	**49.7**	**42.1^#^**	**52.0**	39.5*
Child has a lot to carry^§^	0.57 (75.9%)^‡^	**18.6**	8.0**	19.9	13.8
Driving child to school is more convenient^§^	0.44 (65.4%)^‡^	**42.2**	23.9***	**45.6**	22.7***
It is irresponsible to let children cycle to school with other children, without adult^§a^	0.71^ж^	**9.3**	**5.1^#^**	**15.3**	6.9*
Disapproval from others^§^	0.53^ж^	**3.8**	**0.7^#^**	4.8	1.5
Perceives child's preference is to cycle^§^	0.60 (80.9%)^‡^	**35.8**	75.8***	**20.4**	70.0***
Confident in child's ability to cycle without adult^§a^	0.67^ж^	**73.2**	97.8***	**69.3**	91.1***
The school encourages students to cycle to school^§^	0.71 (80.5%)^‡^	**90.2**	**95.2^#^**	90.9	92.9
***Child perceptions***					
Peer support^¥a^	0.80^ж^	**45.4**	63.1***	**27.4**	41.8**
My school would like students to cycle to school^¥a^	0.45^ж^	**53.8**	**62.0^#^**	55.2	54.9
I have many friends in my neighborhood^¥^	0.76 (87.9%)^‡^	**69.6**	77.7*	**66.2**	**75.8^#^**
**Physical environmental factors**^d^					
***Parent perceptions***					
Neighborhood is safe enough for children to cycle to school with friends^§a^	0.62^ж^	**60.2**	80.4***	**63.0**	81.3**
There are steep hills^§^	0.69 (86.8%)^‡^	10.6	8.0	**12.7**	3.4*
My child would have to cross a busy road^§^	0.68 (83.1%)^‡^	**46.9**	27.8***	**43.3**	21.8***
There are no safe crossings for my child to use^§^	0.69 (81.9%)^‡^	**30.2**	21.0*	**30.9**	16.1**
There is a lot of traffic near the school^§^	0.65^ж^	**53.6**	36.9***	**52.3**	35.6**
Drivers near school often exceed the speed limit^§a^	0.64^ж^	65.6	64.8	**63.5**	**53.9^#^**
***Child perceptions ***					
There are safe places to leave bikes at my school^¥a^	0.74^ж^	**74.9**	**82.1^#^**	79.7	86.8
I would have to cross a busy road^¥a^	0.45^ж^	48.2	50.8	**44.5**	59.3**
I feel safe crossing the road near my school^¥a^	0.56^ж^	**74.1**	82.7*	72.7	72.5
***Objective Environmental factors ***					
School walkability index	-				
High		**45.4**	45.8**	47.5	51.6
Low		**54.6**	**54.2**	52.5	48.4
Road traffic volume	-				
High		**54.6**	54.2**	52.5	48.4
Low		**45.4**	**45.8**	47.5	51.6
Pedshed	-				
High connectivity		**50.5**	51.4*	**54.4**	**63.7^#^**
Low connectivity		**49.5**	**48.6**	**45.6**	**36.3**
Distance (km)					
Mean [SE]		**2.0[0.2]**	1.2[0.1]*** 1.6[0.1] 1.3[0.2]*		

SE = standard error, km = kilometer ^#^*p *≤ 0.10 *p ≤ 0.05 **p ≤ 0.01 ***p ≤ 0.001, ^‡^Kappa (% agreement), ^¥^Item based on child self-report, ^§^Item based on parent self-report, ^ж^ICC, ^Φ^Reliability based on how items were originally measured not on how recoded for analysis.

^a^Measured on a 5-point Likert scale coded 1 = strongly disagree and 5 = strongly agree then recoded into two categories: disagree (disagree, strongly disagree or neither) or agree (agree, strongly agree)

^b^Social factors with no significant bivariate association with cycling to school included the following three items: child's fear of stranger danger; parent perception that other parents in their child's grade allow their child to cycle to school without an adult; whether the parent often sees/hears news items promoting cycling or walking

^c^Measured on a 5-point Likert scale coded 1 = not at all fearful and 5 = extremely fearful then recoded into two categories: not fearful (not at all fearful, not very fearful, somewhat fearful) or fearful (very fearful, extremely fearful).

^d^Perceived environmental factors with no significant bivariate association with cycling to school included the following five items: child perceived neighborhood friendliness, heavy traffic around the school, heavy traffic around the neighborhood; parent perceived lack of footpaths and neighborhood friendliness

After full adjustment (Model 4, Table 3), boys had lower odds of cycling to school the further away they lived, if their parent perceived they had a lot to carry and that driving to school was more convenient. Boys had significantly (p ≤ 0.05) higher odds of cycling to school if their neighborhood was characterized by low traffic and high connectivity, the neighborhood was perceived as safe by their parent, they and their parent had confidence in their ability to cycle, their preference was to cycle and they thought cycling was cool.

**Table 3:** Environmental, social and individual variables associated with boys cycling to/from school ≥ 1 trip/week in logistic regression models.

	Model 1 (*Objective environment)*		Model 2 *(Model 1 + perceptions of environment)*		Model 3 *(Model 2 + social factors)*		Model 4 *(Model 3 + individual factors)*	
**Variables**	**OR**	**95% CI**	**OR**	**95% CI**	**OR**	**95% CI**	**OR**	**95% CI**

Distance (km)	0.82	0.71-0.96*	0.88	0.79-0.98*	0.82	0.67-0.99*	0.70	0.63-0.99*
Low road traffic volume (RTV)^a^	0.20	0.04-0.98*	0.24	0.05-1.24	0.52	0.11-1.50	0.66	0.07-1.14
High pedshed (high connectivity)^b^	0.60	0.26-1.37	0.72	0.23-2.22	0.50	0.18-0.95	0.35	0.22-1.17
Low RTV^x^high pedshed^#^	8.04	1.36-47.56*	6.19	0.86-44.57	4.83	1.09-21.50*	5.58	1.11-27.96*
Neighborhood is safe enough for children to cycle to school with friends^‡c^			2.39	1.57-3.64***	1.64	0.99-2.72	1.74	1.08-2.80*
My child would have to cross a busy road^‡d^			0.51	1.41-2.79***	0.68	0.48-0.96*	0.77	0.47-1.27
Child has a lot to carry^‡d^					0.50	0.27-0.93*	0.43	0.21-0.89*
Driving child to school is more convenient^‡d^					0.51	0.31-0.85*	0.42	0.23-0.74**
Perceives child's preference is to cycle^‡e^					7.58	3.99-14.42***	5.08	2.62-9.87***
Confident in child's ability to cycle without adult^‡c^					10.60	3.85-29.25***	10.39	3.79-28.48***
Cycling is child's preference^e^							5.68	3.23-9.98***
Cycling to school is cool^c^							1.85	1.19-2.88**
Confident in ability to cycle to school without adult^c^							3.42	1.30-9.00*

RTV = road traffic volume, OR = odds ratio, CI = confidence interval, *p ≤ 0.05 **p ≤ 0.01 ***p ≤ 0.001,^‡^Parent perception, ^#^Interaction term. Reference categories: ^a^High RTV ^b^Low pedshed ^c^Strongly disagree/disagree/neither, ^d^Disagree/not applicable, ^e^Yes vs. No

All models adjusted for school clustering, school grade and maternal education.

After full adjustment (Model 4, Table 4), the odds of cycling to/from school were lower among girls whose parents perceived they had to cross a busy road, whose parent considered driving to school more convenient and who had an adult home every day after school. The odds of cycling to/from school were greater if their preference was to cycle and both parent and child had confidence in their ability to cycle.

**Table 4:** Environmental, social and individual variables associated with girls cycling to/from school ≥ 1 trip/week in logistic regression models.

	Model 1 (*Objective environment)*		Model 2 *(Model 1 + perceptions of environment)*		Model 3 *(Model 2 + social factors)*		Model 4 *(Model 3 + individual factors)*	
**Variables**	**OR**	**95% CI**	**OR**	**95% CI**	**OR**	**95% CI**	**OR**	**95% CI**

Neighborhood is safe enough for children to cycle to school with friends^‡a^			2.21	1.16-4.24**	1.72	0.89-3.32	1.73	0.89-3.38
My child would have to cross a busy road^‡b^			0.32	0.19-0.56***	0.37	0.23-0.61***	0.44	0.25-0.76**
I would have to cross a busy road^a^			0.42	0.23-0.78**	0.55	0.30-1.01	0.59	0.30-1.15
Driving child to school is more convenient^‡b^					0.44	0.23-0.83**	0.40	0.20-0.82**
Perceives child's preference is to cycle^‡c^					10.18	5.30-19.55***	7.69	3.77-15.66***
Confident in child's ability to cycle without adult^‡a^					3.63	1.93-6.82***	4.03	2.02-8.05***
Adult home after school on most days^c^							0.41	0.20-0.83**
Cycling is child's preference^c^							3.73	2.26-6.17***
Confident in ability to cycle to school without adult^a^							2.13	1.29-3.52**

OR = odds ratio, CI = confidence interval, *p ≤ 0.05 **p ≤ 0.01 ***p ≤ 0.001,^‡^Parent perception

Reference categories: ^a^Strongly disagree/disagree/neither, ^b^Disagree/not applicable, ^c^Yes vs. No

All models have been adjusted for school clustering, school grade and maternal education

**Figure 1:**
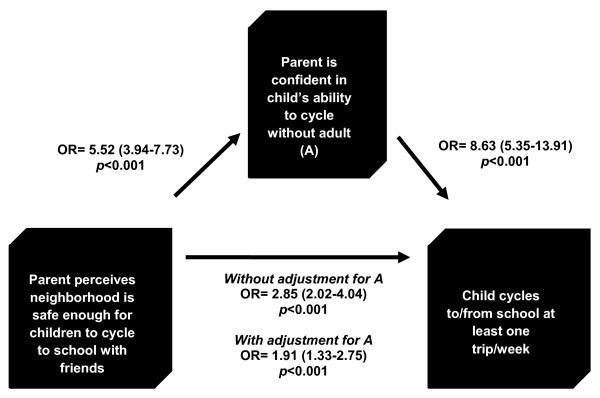
**Relationship between parent perceived neighborhood safety, parent confidence in child's cycling ability and cycling to/from school ≥ 1 trip/week, for boys and girls**.

For both boys and girls, the strength of the association between neighborhood safety and cycling attenuated and became non-significant once social factors were added to the model 3, (Tables 3, 4). As shown in Figure 1, mediation analysis [24] that combined boys and girls showed that the association between parental perception of neighborhood safety and cycling to or from school was partially mediated by parent's confidence in their child's ability to cycle to school without an adult, as there was a reduction in the regression coefficient after adjusting for parental confidence in their child's ability. Moreover, urban features were associated with whether parents perceived the neighborhood to be safe enough to allow their child to cycle to school without an adult and how confident parents were in their child's ability to cycle safely to school without an adult. For example, if the school neighborhood was characterized by low traffic and high connectivity, the odds of parents perceiving the neighborhood as safe (OR: 3.50, 95% CI 1.18-10.36) and having confidence in their child's ability to cycle were higher (OR: 2.15, 95% CI 1.08-4.26). For every 1 km the child lived from the school, the odds that parents perceived the neighborhood to be safe decreased by 0.87 (95% CI 0.81-0.93).

## Discussion

This study used an ecological framework to examine individual, social and environmental factors associated with cycling to school, stratified by gender and is one of the first to consider cycling independent of other forms of active transport. Urban features such as distance, traffic exposure and pedestrian connectivity were associated with cycling behavior in boys, however in girls, parental perceptions of the environment appeared more important. These results support other evidence within the AST literature that distance and parental concerns about traffic safety are associated with AST [20,25-27] and emphasize the importance of proximate school catchment areas with highly connected streets and low traffic volumes to encourage cycling to school [28,29].

Environmental perceptions regarding neighborhood safety issues (i.e., whether the neighborhood is safe enough and the need to cross busy roads) were associated with cycling in boys and girls. Safety is a common correlate of AST [11,13,16,27]. Kerr and colleagues [26] found that children whose parents had few safety concerns were up to five times more likely to use AST compared with parents who had more concern [26]. However, they argue that a simple interpretation of this association, that parental education could increase children's active transport, should be resisted because parental concerns were related to real safety issues such as poor walking and cycling facilities and traffic danger. Interventions that attempt to change parental perceptions without considering the commuting environment may be less effective. Approaches such as the Safe Routes to School interventions that aim to improve safety through planning and design [30] in combination with promotion activities [31] hold most promise.

This study found that parental confidence in their child's ability to cycle to school mediated the association between perceived safety and cycling. This highlights that in addition to modifying the environment to make it safer, skill development may also be an important strategy to help alleviate parental concerns about safety and increase cycling. Cycling accidents are among the most common causes of physical injury to children [32], and a number of studies have found that untrained [33] or less cycling-proficient children [34] have much higher accident rates than other children, even though they may cycle less frequently. Thus, educational programs that develop children's motor development, such as pedaling, balancing, steering, and braking as well as cognitive elements such as concentration, attention, judgment, planning, decision making and overall confidence are likely to be important [35]. Moreover, education of the road rules, wearing the right protective gear and bicycle maintenance are other important skills needed to make children's journey to school safer [32] and increase parent's confidence in their child's ability to negotiate their environment.

This study also found that if parents perceived driving their child to school was more convenient, the likelihood of cycling in both boys and girls was significantly decreased. Indeed, previous AST research has found that parents are more likely to perceive car travel as more convenient than walking or cycling [12]. Lorenc and colleagues suggest that parents' emphasis on the convenience of car travel may relate to cultural influences, e.g. the perception that walking and cycling are associated with low social status [12]. It may also be a reflection of private cars becoming the solution for busy time-poor households fulfilling scheduling commitments by linking school travel with other activities. Clearly, strategies to combat this aspect of children's increasing car travel are probably the most challenging as they are very much dependent upon the individual household's structure, decision-making and lifestyle choices. Nevertheless, behavior change programs are likely to be more successful if they are undertaken in conjunction with environmental interventions to make walking and cycling more convenient. For example, Morris and colleagues suggest co-locating schools with facilities where afterschool activities are conducted (e.g., community centers and sporting fields) may assist in providing children with the option of walking or cycling while at the same time reducing the demands on parents' time [36].

The cross-sectional design, lack of information on non-respondents and limited range of objective environmental variables assessed in the study are limitations. The school-specific walkability index was not based on cyclability and it is possible the environmental correlates may be stronger if explicit environmental measures of the presence and quality of cycling infrastructure were measured. Distance to school may not be accurate because potential 'access' points generated around each school boundary may not reflect true access points, the shortest route may not be the route actually taken [27] and digitization of the informal pedestrian network may have missed some potential cut-throughs and paths through parks. Since few studies have specifically examined cycling to school, most survey items were newly developed or modified from existing items regarding walking to school. However, we did undertake test-retest reliability and internal reliability testing. Furthermore, other approaches to the analysis of this study could have been undertaken. For example, analyses could have been stratified by proximity to school which may have provided further insights into the differences in correlates of cycling to school and an alternative modeling strategy based on theoretical significance rather than statistical significance could have been adopted. Future studies might also like to use more advanced statistical methods (e.g. structural equation modeling) to explore other mediation pathways. Despite these limitations, the study included a relatively large sample, used an ecological approach, included a combination of objective and perceived measures of the environment and stratified the analysis by gender.

## Conclusions

In conclusion, our results suggest that there is great potential for increasing levels of cycling to school in children. Creating child cycle-friendly communities while at the same time providing children with the skills to safely navigate the environment are important strategies required to increase cycling to school and give parents the confidence that their child can safely do so. The results suggest that urban planning strategies aimed at promoting more child cyclable neighborhoods might focus on ensuring residential areas are proximate to schools and improving street connectivity and reducing traffic volumes around schools. This highlights the importance of policies concerned with where schools are situated and the traffic carried by roads in the school catchment area. Initiatives clearly need to target parents as well as children and programs such as bicycle education should focus on providing educational and practical support to ensure safe journeys to school. Given the lack of empirical knowledge about factors related to cycling to school, it is important that future studies seek to further understand the role of individual, social and environmental factors in order to design effective interventions in different settings.

## List of Abbreviations

(AST): active school transport; (95% CI): 95 percent confidence interval; (OR): odds ratio; (TREK): Transport, Environment and Kids project; (GIS): Geographic Information Systems; (RTV): road traffic volume; (SWI): school-specific walkability index; (SES): socioeconomic status; (RR): response rate; (ICC): intraclass correlation; (BMI): body mass index; (SD): standard deviation; (SE): standard error.

## Competing interests

The authors declare that they have no competing interests.

## Authors' contributions

GSAT conceived of and drafted the manuscript. All authors except HEC were involved in the TREK data collection effort, BGC, MB, AT and GRM were supervisors and CI's on the grant, GSAT was the Project Co-ordinator, and KV was a Research Assistant. All authors contributed to interpretation and writing of the manuscript. All authors read and approved the final manuscript.
